# Spatio-temporal patterns of the oceanic conditions and nearshore marine community in the Mid-Atlantic Bight (New Jersey, USA)

**DOI:** 10.7717/peerj.7927

**Published:** 2019-10-21

**Authors:** Juan C. Levesque

**Affiliations:** 1 Earth and Environmental Science, University of Texas at Arlington, Arlington, TX, United States of America; 2 Environmental Resources Management, Tampa, FL, United States of America

**Keywords:** Climate variability, Community dynamics, Ecosystem science, Experimental design, Fisheries independent monitoring, Fisheries and fish science, Population dynamics

## Abstract

Oceanic environmental conditions influence, shape, and control the geographical range, spatial distribution, abundance, and size composition of marine fauna. Water temperature, salinity, dissolved oxygen, depth, and sediment type influence select fish life-history characteristics and community structure. Marine communities are vulnerable to major changes in environmental conditions, but the response and severity depends on various biological or ecological factors, such as resilience to stress or adaptation. Researchers around the world have predicted and documented numerous alterations in fish communities caused by ongoing significant physicochemical shifts associated with natural and potentially unnatural sources, but published studies describing the historical conditions are lacking for most regions around the world, including the coastal waters off New Jersey. Given the need to understand these processes, a multifaceted investigation was undertaken to describe, evaluate, and compare the oceanic conditions and nearshore marine fauna community off New Jersey during 1988 through 2015. Findings showed the oceanic conditions varied over time and space. Mean surface water temperature increased significantly about 0.6 °C per decade, mean salinity decreased about 1.3 psu per decade, and dissolved oxygen increased 0.09 mg/l per decade. Over 20.4 million fish and invertebrates (1,338.3 mt) representing 214 (water temperature preference classified) species (*not including unidentified species*) were collected within 15 strata (areas: 12−26) off the coast of New Jersey from 1988 to 2015. Three marine fauna water temperature preference groups (coldwater-adapted, warmwater-adapted, and subtropic-adapted) were identified in the study area. The main coldwater-adapted species collected were longfin squid (*Loligo pealei*) (*n* = 2, 225, 975), Atlantic herring (*Clupea harengus*) (*n* = 544, 032), and little skate (*Leucoraja erinacea*) (*n* = 316, 356), while Atlantic butterfish (*Peprilus triacanthus*) (*n* = 2, 873, 138), scup (*Stenotomus chrysops*) (*n* = 1, 318, 569), and northern searobin (*Prionotus carolinus*) (*n* = 503, 230) represented the warmwater-adapted group. Bay anchovy (*Anchoa mitchilli*) (*n* = 9, 227, 960), striped anchovy (*Anchoa hepsetus*) (*n* = 245, 214), and Atlantic moonfish (*Vomer setapinnis*) (*n* = 38, 691) denoted the subtropic-adapted group. Subtropic-adapted species were the most abundant and coldwater-adapted were the least abundant water temperature preference group. The estimated abundance of coldwater-adapted species declined, warmwater-adapted species slightly increased, and subtropic-adapted species decreased with time, which suggest the environmental conditions are influencing and thereby shifting the marine community.

## Introduction

Oceanic environmental conditions influence, form, and control the geographical range, spatial distribution, abundance, and size composition of marine fauna. Fish life-history and community structure characteristics are shaped by water temperature, salinity, dissolved oxygen (DO), depth, and sediment type (Horne & Campana, 1989). For many open-water coastal species (non-estuary dependent), water temperature is usually the most important environmental factor influencing fish distribution (e.g.,  Hoese & Moore, 1977; Wood, Collie & Hare, 2009; Howell & Auster, 2012). Based on a species’ physiology, marine fauna have an optimal temperature range that effect their behavior, distribution, abundance, and other life-history characteristics. In most regions, water temperature varies with season, which influences migratory behavior (Parker Jr & Dixon, 1998).

In addition to effecting the distribution, mean size, and life span (Muyodi, Mwanuzi & Kapiyo, 2011) of fishes, fluctuations in the physicochemical conditions can also impact the regional community structure (Reash & Pigg, 1990; Vinebrooke et al., 2004; Krishnakumar & Bhat, 2008), and the associated food-chain length (e.g., Bondavalli et al., 2006). According to Ficke, Myrick & Hansen (2007), a major change in the environment conditions causes fish to either “adapt, migrate, or perish”. Marine communities are vulnerable to changes in environmental conditions, which can have direct and indirect impacts.

Researchers worldwide have predicted and documented numerous changes in marine communities caused by ongoing physicochemical shifts associated with natural and potentially unnatural sources (Rijnsdorp et al., 2009; Crozier & Hutchings, 2014; Pinsky & Mantua, 2014), but fundamental information describing marine communities are lacking for most regions (Johnson, 2001), including the nearshore waters off New Jersey; New Jersey is located within the middle or Mid-Atlantic Bight (MAB) in the western North Atlantic Ocean.

In spite of the economic value of New Jersey’s fisheries resources, only partial information about the nearshore marine community and environmental conditions is available. As such, the main goal of this study was to elucidate the trends in the environmental conditions and the nearshore marine fauna community off New Jersey over the past 28 years (1988–2015). The primary purpose was to provide resource managers and others with a description of the environmental conditions and marine fauna in the nearshore waters off New Jersey so they can make knowledgeable management decisions about marine resources, predict future changes in populations, and potentially reconstruct the past historical baseline conditions given the shifting baseline syndrome. Establishing the “relative” baseline conditions will help resource managers and researchers evaluate potential future impacts to the biological community associated with natural and anthropogenic disturbances in the nearshore waters off New Jersey.

## Material and Methods

### Study area and survey design

**Figure 1:**
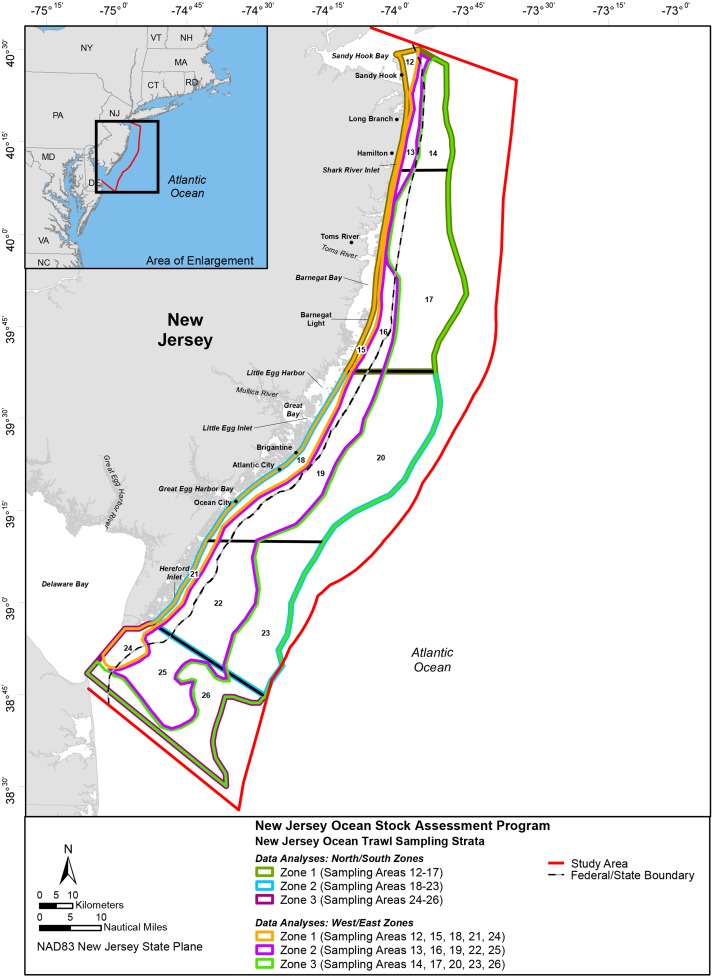
Study Area; New Jersey Ocean Stock Assessment Program. The colored lines depict different zones (Figure credit: Casey Gomez 2019).

To evaluate marine community dynamics, the survey area (Fig. 1) was divided into 15 sampling strata (12–26) by depth and geographical location (latitude and longitude). To be consistent with established federal marine resource field-sampling programs, the designers of the New Jersey Ocean Stock Assessment (OSA) survey incorporated the same latitudinal boundaries defined by the National Marine Fisheries Service (NMFS), Northeast Fishery Science Center (NEFSC), Northeast Atlantic Groundfish Survey Program; exceptions were those strata at the northern and southern ends of the New Jersey coastline where NMFS extended its survey into New York and Delaware waters (ASMFC, 1994). The boundaries were also truncated in the northern and southern strata to include only the waters adjacent to the New Jersey coastline and the ocean waters off Delaware Bay. The longitudinal boundaries consisted of the 9.1 (30 ft.), 18.3 (60 ft.), and 27.4 m (90 ft.) isobaths. The bottom contours were somewhat irregular, so the stratum boundaries were smoothed using GIS techniques (ASMFC, 1994).

To reduce potential sampling bias, each sampling area was divided into smaller blocks. Mid-shore blocks (9.1–18.3 m) and offshore (18.3–27.4 m) blocks were 2.0 min longitude by 2.5 min latitude, whereas nearshore (5.5–9.1 m) blocks were 1.0 min longitude by 1.0 min latitude. Nearshore block dimensions were smaller because the strata were narrower and encompassed a smaller area than the mid and offshore strata; thus, the smaller block size permitted a greater number of potential sampling sites than would be possible with larger dimensions. It should be noted the blocks truncated by stratum boundaries encompassed a smaller area (>50%) than the whole blocks (Byrne, 1994; Byrne, 2008).

### Experimental field sampling approach

Field sampling was conducted bimonthly (every two months: February, April, June, August, October, and December) from 1988 to 1989. From 1990 to date, the December and February surveys were replaced by a single winter survey in January, followed by surveys in April, June, August, and October (ASMFC, 1994) The annual sampling survey effort during 1988 through 1990 varied slightly because of the limited budget (e.g., high charter vessel costs), but annual sampling generally consisted of two hauls per stratum. Overall, the sampling effort averaged 39 hauls (i.e., two samples from each strata plus one additional haul in each of the nine larger strata) per survey. The average number of stations sampled each year was around 182.

### Station selection

Constrained randomization was used to select unique sampling stations for each survey trip. Sampling stations (survey site location) were randomly selected by the NJDEP program leader during 1988 through 1991, but this method was replaced in 1992 by a computer generated random number selection program. Because stratum shapes were elongate and the sampling effort was limited, a station selection procedure was used to reduce any spatial distribution sampling bias. The station selection procedure consisted of limiting the first station to only the top half of the block numbers and the second station to the bottom half; however, if a third station was selected then these limitations were not imposed in the procedure process. For instance, haul one would be selected from blocks 1 to 25, haul two from blocks 26 to 50, and haul three from blocks 1 to 50 for a stratum with 50 blocks. For each station, three additional alternate sites were also pre-selected using the same procedures described above to account for any fixed fishing gear (e.g., traps or nets), bottom obstructions, or other impediments that prevented sampling at the initial station Byrne, 1994; Byrne, 2008.

### Field sampling gear

Field sampling was conducted with a tapered (forward to rear netting) three-in-one bottom otter trawl. The forward (i.e., wings and belly) and rear netting was constructed with #3/20 twisted polyethylene twine. The forward and rear netting was constructed with 12 cm and eight cm stretch mesh, respectively. The cod-end was constructed with 7.6 cm stretch mesh four mm polyethylene twine and lined with #147 style (6.4 mm stretch mesh) white knitted nylon netting. The round and drop-mesh corners were hung to yield a 50 percent hanging ratio. The trawl doors were 2.44 m × 1.27 m (3.1 m^2^) constructed with marine grade wood and steel shoes that weighed approximately 453.5 kg. Based on hydroacoustic sensors, the average wing spread was around 13 m. The headrope was 25 m and the footrope was 30.5 m. The top and bottom bridles were 36.6 m and constructed with a 1.27 and 1.91 cm wire rope, respectively. The groundwire was 18.3 m. The bottom bridle and groundwire were covered with 6.03 cm rubber cookies. The rigging (sweep) consisted of 7.62 cm rubber cookies on 14.3 mm wire rope, 9.5 mm long drop chains (six link long), and 32 center-hole floats (20.32 cm). The extension chain was a 1.27 cm Trawlex chain Byrne, 1994; Byrne, 2008.

### Field sampling procedures

All tows were conducted between sunrise and sunset. The trawl tow duration was standardized at 20 min and the vessel speed was between 4.7 and 5.6 kilometers per hour (2.5 and 3.0 knots). The swept area (a) was estimated with the following equation: *a* = *D*∗*hr*∗*X*2, *D* = *V*∗*t*; where *V* is the velocity of the trawl over the ground when trawling, hr is the length of the head-rope, and t is the time spent trawling. X2 is that fraction of the head-rope length, hr, which is equal to the width of the path swept by the trawl, the “wing spread”, *hr*∗*X*2. Based on vessel speed, one 20 min tow generally covered a distance of 1.85 km. Given the trawl dimensions and distance towed, the total swept area was around 24,050 m^2^. The cardinal direction of the tow was determined by the oceanic conditions (wind, waves, and current) at the time of deployment; tows were generally made in the direction of the waves, wind, and current. A 91.5 m wire was used to maintain a tow depth ratio of approximately 3:1 Byrne, 1994; Byrne, 2008. Survey replicates (tow) were considered independent of one another given the random station selection process, and the distance between sampling sites and time between tows; each tow was considered a random sample of the population.

### Data collection protocol

At each sampling station the surface and bottom environmental conditions (water temperature (degrees Celsius (°C), salinity (parts per thousand [psu], and dissolved oxygen (milligrams per liter (mg/l)) were measured with a CTD and recorded before deploying the trawl. After the 20 min tow was completed, the trawl was retrieved and the catch (fish and macroinvertebrates) was rough sorted into plastic buckets. Afterwards, the entire catch was identified to species, enumerated, and the length (fork and/or total, as appropriate) was measured to the nearest cm for fish (20 individuals randomly selected); the disk width (cm) was measured for skates and rays. All species were identified to the lowest taxa. The total weight was taken using either a hanging or floor scale. The individual weights of every species were determined by weighing individual baskets (total weight) of every species collected and dividing by the total count of individuals in the basket. Various other measurements were recorded depending on the macroinvertebrate species. For example, the carapace width (mm) was measured for crabs, the carapace length (mm) for lobsters and mantle length (mm) for squids. Because some catches were too large to sort in the field, a representative thoroughly mixed sub-sample was randomly selected and weighed. After the sub-sample was sorted, species composition was extrapolated to determine the total catch (Byrne, 1994; Byrne, 2008).

### Data treatment/processing

To minimize any potential spatial non-independence, data were pooled among stations within each individual sampling area. Before initiating statistical hypothesis tests, environmental and biological data were transformed (e.g., logarithmic, square root, fourth root, or arcsine) to meet normality assumptions, and down-weight the statistical effects (i.e., reduce skewness) of abundant taxa, while allowing less common taxa to contribute to sample discernment (Thorne, Williams & Cao, 1999; Korsman, 2013). Normal probability plots were examined, and Kolmogorov–Smirnov and Bartlett tests were used to assess normality and homoscedacity (Zar, 1999). Outlier observations were investigated to determine whether the outlier occurred by chance; all outliers were retained for these analyses.

To evaluate the nearshore marine community and oceanic conditions, 28 years (1988–2015) of fishery-independent monitoring data (environmental and biological) were compiled, sorted (time [year and month] and space [area and zone]), and summarized. After pooling the data by stations sampled within each area, the marine community (catch characteristics [total number, estimated abundance, and estimated biomass]) was evaluated using two approaches: a single dataset (pooling all the data) and segregating the data in various time-series datasets. The data were segregated into six 5-year time-series periods to help discern patterns and test for potential differences among time and space. This approach was driven from the perspective that most available time-series data for nearshore/offshore fisheries are only two to five years in duration. Data were pooled by stations and segregated by individual sampling areas (12–26) and geographical north/south zones defined as the following: 1 (sampling areas 12–17), 2 (sampling areas 18–23), and 3 (sampling areas 24–26). Data was also segregated by west/east zones defined as the following: 1 (sampling areas 12, 15, 18, 21, and 24), 2 (sampling areas 13, 16, 19, 22, and 25), and 3 (sampling areas 14, 17, 20, 23, and 26). It should be noted the amount of area for each designated geographical zone was a different size in terms of km^2^.

Following Howell & Auster (2012), marine species were classified a priori as coldwater-adapted species (primarily distributed in cold temperate regions), warmwater-adapted species (primarily distributed in warm temperate regions), or subtropic-adapted species (primarily distributed in subtropical and tropical regions). Classification followed Froese & Pauly (2018) and published life-history literature (e.g.,  Murdy, Baker & Musick, 1997; Collette & Klein-MacPhee, 2002; Able & Fahay, 2010) describing a species’ distribution relative to the MAB, water temperature tolerance (minimum and maximum), preferred water temperature range, and preferred spawning water temperature. In general, the mean preferred water temperature was used to select the best water temperature preference group for each species. Species preferring water temperature <15 °C were generally classified as coldwater-adapted, while those preferring water temperatures 15–29 °C were classified as warmwater-adapted. Species preferring temperatures >30 °C were classified as subtropic-adapted.

The statistical significance level was defined as *P* < 0.05. In the presence of significance at the 95 percent confidence level, a Tukey *post-hoc* multiple pairwise comparison tests were used to differentiate the specific differences among the population means. Data were evaluated using various software, including Microsoft Access^®^, Microsoft Excel^®^, and Statgraphics Centurion XVI^®^.

### Statistical analyses

#### Physicochemical conditions

A two-fold approach was taken to analyzing variation over time. Interannual variation of numerous factors was examined by treating observations from individual stations and months as independent, generating a sensitive Analysis of Variance (ANOVA) test of the null hypothesis of no variation due to the high degrees of freedom. To examine trends, annual averages were regressed against time (year), generating a conservative test with low degrees of freedom and a weaker independence assumption of no serial correlation between annual averages. The physicochemical conditions were evaluated by univariate procedures to discern patterns over space and time. Descriptive statistics and graphical plots were generated for each individual defined sampling area (12–26; Fig. 1). Student’s *t*-tests were used to test the null hypothesis that the annual average surface and bottom oceanic conditions (water temperature, salinity, and dissolved oxygen (DO)) were equal among years (1988–2015). One-way ANOVAs were used to test the null hypothesis that annual and bimonthly oceanic conditions (water temperature, salinity, and DO) were equal among years and sampling areas. To characterize the physicochemical conditions within the study area (1988–2015), the annual mean surface and bottom water temperature, salinity, and DO readings were individually examined for spatial and interannual patterns using linear regression to categorize the trend as stable, increasing, or decreasing. The strength of the association was examined using the coefficient of determination (r^2^). One-way ANOVAs were also used to test the null hypothesis that annual and bimonthly bottom oceanic conditions (water temperature, salinity, and DO) were equal among zones and depth boundaries. Regression was also used to evaluate the association between space (latitude and longitude) and time.

#### Marine Community

To evaluate the biological patterns and trends, the marine community (catch characteristics [total number, estimated abundance, and estimated biomass]) was examined using various univariate procedures. The total number marine fauna collected by individual taxa were tabulated, summarized, and plotted by time and space. Descriptive statistics, histograms, frequency distribution, and cumulative frequency polygon plots were generated to evaluate central tendency, dispersion, and variability. To evaluate seasonal (bimonthly) and annual variability in the estimated abundance (density (number of fauna collected per 100 m^2^)), the total number of individuals collected by species were standardized, transformed into nominal catch per unit effort (CPUE) indices, and evaluated using several analytical approaches. For analyses and interpretation of the abundance indices, it was assumed there was a simple direct positive relationship between CPUE and abundance. To estimate abundance as a function of effort, CPUE was calculated by taking the product of the area swept, which was computed from the trawl net width at the wingtips and the distance towed; the trawl wing or horizontal spread was determined using hydroacoustic sensors. Abundance (*N*_*t*_ number per 100 m^2^) was estimated using the CPUE, the trawl dimensions, and the vessel speed in the following equation:

\begin{eqnarray*}{N}_{t}= \frac{C}{AL} \times 100 \end{eqnarray*}

where *C* is catch of species (*i*) at time _*t*_, *A* is the mouth area of the trawl (24,076 m^2^), and *L* is the distance towed (∼1.85 km), which was the product of the vessel speed (92.5 m s^−1^) and the trawl time (20 min). To estimate biomass (g 100 m^−3^), *W* (catch in weight) was substituted for *C* in each tow.

The annual estimated abundance and biomass index (mean number/weight per tow) were computed, compared, and regressed over the 28-year time series to examine change in estimated abundance and biomass over time and space. To examine annual variability in species composition, ANOVA tests were conducted to test the null hypothesis that the total number, estimated abundance and biomass were equal over time and space. Regression was also used to examine the association between catch characteristics and time and space using the fitted slope to indicate increasing or decreasing trends.

Biological data (abundance and biomass) were segregated by individual sampling area (12–26) and geographical zones. North/south zones were defined as the following: 1 (sampling areas 12–17), 2 (sampling areas 18–23), and 3 (sampling areas 24–26). West/east zones were defined as the following: 1 (sampling areas 12, 15, 18, 21, and 24), 2 (sampling areas 13, 16, 19, 22, and 25), and 3 (sampling areas 14, 17, 20, 23, and 26). Regression was used to evaluate the association between space (latitude and longitude) and time. One-way ANOVAs were used to test the null hypothesis that annual and bimonthly catch characteristics (total number, estimated abundance and biomass) were equal among zone and depth boundary. Spatial and temporal patterns were evaluated using regression to categorize the slope of the fitted trend.

Descriptive statistics were generated to examine the number of warmwater, coldwater, and subtropic-adapted species. ANOVAs were used to test the null hypothesis that the catch characteristics (total number, estimated abundance and biomass) by temperature preference category were equal over time and space. Descriptive statistics were also generated to evaluate the ratio of warmwater to coldwater-adapted species. Spatial and temporal patterns were evaluated using regression to categorize the slope of the fitted trend.

Individual descriptive statistics of the catch, and temperature preference category were calculated and plotted by time-series. To examine annual variability in the marine fauna, separate ANOVA tests were conducted to test the null hypothesis that the catch characteristics ((total number, estimated abundance, and biomass)) were equal among time-series, month, and area. Two-way ANOVA tests were conducted to test the null hypothesis that the catch characteristics ((total number, estimated abundance, and biomass)) by individual temperature preference category were equal among time and space. Regression was used to examine the potential association between catch characteristics and time-series, month, and area. Spatial and temporal patterns were evaluated using regression to categorize the slope of the fitted trend. General Linear Models (GLM) were calculated for each time-series (overall and temperature preference category) to examine the pattern of interactions and associations of time and space on the catch characteristics.

## Results

### Physicochemical conditions

#### Water temperature

The overall mean annual surface water temperature off the coast of New Jersey within the 15 strata (areas: 12–26) during 1988 through 2015 ranged from 13.39 °C in 2003 to 16.12 °C in 2002 with a mean of 14.81 °C (±6.6 °C). A paired *t*-test showed the mean surface water temperature was significantly warmer (2.3 °C) than the bottom water temperature (*t* (5096) = 3.72; *P* < 0.05). The mean surface water temperature varied significantly among years (ANOVA, Table 1), and there was a weak positive association between the surface water temperature and time. The mean (0.06 °C per year) and maximum (0.02 °C per year) surface water temperature increased about 0.6 °C and 0.2 °C per decade, respectively. Averaging over years, the mean monthly surface water temperature increased from January (4.46 °C) to August (22.74 °C), and decreased from September (22.24 °C) to December (5.65 °C). The surface water temperature varied significantly among months (*F* [11, 5084] = 5,942.1, *P* < 0.05).

**Table 1:** The annual physicochemical conditions in the study area (1988−2015).

**Environmental parameter**	**Hypothesis test for interannual variation**	**Test results**	**Regression model**	**Regression equation for time trend**	*F*-test	*r*^**2**^
Surface water temperature	ANOVA	*F* [26, 5068] = 3.13, *P* < 0.05)	Linear	Surface Temperature = −37.0457 + 0.0259257*Year	*F* [1, 26] = 1.77, *P* = 0.1945	6.4%
Bottom Water Temperature	ANOVA	*F* [26, 5068] = 6.35, *P*= <0.05	Linear	Bottom Temperature = −22.9444 + 0.0177299*Year	*F* [1, 26] = 0.54, *P* = 0.4678	2.0%
Surface Salinity	ANOVA	*F* [26, 5068] = 18.97, *P* < 0.05	Linear	Surface Salinity = 90.1532 − 0.0296713*Year	*F* [1, 26] = 4.20, *P* = 0.0505	13.9%
Bottom salinity	ANOVA	*F* [26, 5069] = 26.97, *P* < 0.05	Linear	Bottom Salinity = 90.0402 − 0.0291552*Year	*F* [1, 26] = 6.87, *P* = 0.0144	20.9%
Surface dissolved oxygen	ANOVA	*F* [26, 5066] = 5.20, *P* < 0.05	Linear	Surface DO = -10.5943 + 0.0095525*Year	*F* [1, 26] = 2.74, *P* = 0.11	9.5%
Bottom dissolved oxygen	ANOVA	*F* [26, 5067] = 6.42, *P* < 0.05	Linear	Bottom DO = −15.1643 + 0.0113536*Year	*F* [1, 26] = 2.17, *P* = 0.1526	7.8%

The mean surface water temperature ranged from 13.8 °C in sampling area 12 to 15.52 °C in sampling area 23. In general, the mean surface water temperature was colder in the northern than the southern sampling areas (Table S1); the 15 sampling areas were numbered in numerical order from north to south (12–26). However surface water temperature in sampling areas 21 and 24 did not follow this pattern; the surface water temperature in these areas was slightly cooler. The mean water temperature in sampling area 21 was cooler than the water temperature in sampling area 24. A linear model showed a strong positive (significant) association between the mean annual surface water temperature and the sampling area (Table S1).

The mean bottom water temperature off the coast of New Jersey within the 15 strata (areas: 12–26) during 1988 through 2015 ranged from 10.44 °C in 1994 to 14.57 °C in 2002 with a mean of 12.53 °C (±5.6 °C). The bottom water temperature also varied significantly with time, and there was a weak positive association between bottom water temperature and year (Table 1). Averaging over years, the bottom water temperature varied significantly among months (*F* [11, 5084] = 1637.8, *P* < 0.05) with the coldest (4.96 °C) in January and the warmest (20.51 °C) in September.

The mean bottom water temperature ranged from 10.67 °C in sampling area 14 to 14.25 °C in sampling area 24. The mean bottom water temperature varied significantly among sampling areas (Table S2), and there was a weak positive association between bottom water temperature and the sampling area (Table S2). Segregating the sampling areas into zones (*See Methods*), the mean bottom water temperature was significantly colder in the northern zones than the southern zones (*F* [2, 5093] = 23.08, *P* < 0.05); a *post-hoc* test showed the mean water temperature varied significantly between zones 1 and 2 (−0.89), 1 and 3 (−1.36), and 2 and 3 (−0.47). The warmest bottom water temperature was in the sampling areas closest to shore, and the coldest bottom water temperature was detected in sampling areas furthest from shore (*F* [2, 4828] = 42.11, *P* < 0.05); a *post-hoc* test showed the mean water temperature varied significantly between eastern and western zones 1 and 2 (0.52), 1 and 3 (1.74), and 2 and 3 (1.22).

### Salinity

The mean annual surface salinity off the coast of New Jersey within 15 strata (areas: 12–26) during 1988 through 2015 ranged from 29.94 in 1996 to 32.11 psu in 1998 with a mean of 30.75 psu (±2.06 psu). Overall, the mean salinity decreased about 0.13 psu per year or 1.3 psu per decade. The surface salinity varied significantly among years, and there was a weak negative (non-significant) association between the surface salinity and time (Table 1). Averaging over years, the mean monthly surface salinity ranged from 30.25 in April to 31.48 psu in November with a mean of 30.73 psu (±2.07 psu). The surface salinity varied significantly among months (*F* [11, 5084] = 31.46, *P* < 0.05).

The mean surface salinity ranged from 28.92 in sampling area 14 to 31.45 psu in sampling area 26 with a mean of 30.74 psu (±2.07 psu). The surface salinity varied significantly among sampling areas (Table S1), and there was a strong positive (significant) association between the surface salinity and sampling area (Table S2). The surface salinity was generally lower in the northern sampling areas than the southern sampling areas (Table S1 **)**. The surface salinity ranged from 28.13 psu in sampling area 12 to 31.49 psu in sampling area 26. The lowest surface salinity levels were found in the areas closest to shore, and the highest surface salinity levels were detected in areas furthest from shore.

A paired *t*-test showed the mean annual bottom salinity level was higher than the mean annual surface salinity (*t* (5095) = −34.25; *P* < 0.05). The lowest (30.52 psu) bottom salinity was in 2005 and the highest (32.87 psu) was in 1988 with a mean of 31.66 psu (±1.4 psu). The bottom salinity varied significantly with time (*F* [27, 5069] = 50.12, *P* < 0.05), and there was a weak negative (significant) association between the surface salinity and time (Table 1). The bottom salinity ranged from 31.09 in December to 31.94 psu in November. Averaging over years, the bottom salinity also varied significantly among months (*F* [11, 5085] = 5.13, *P* < 0.05).

The mean bottom salinity ranged from 30.33 in sampling area 12 to 32.32 in sampling area 23 with a mean of 31.66 psu (±1.4 psu). The bottom salinity varied significantly among sampling areas (Table S2), and there was a weak negative association between the bottom salinity and the sampling area (Table S2). Segregating the sampling areas into zones (*See Methods*), the mean bottom salinity varied significantly among northern and southern zones (*F* [2, 5094] = 14.31, *P* < 0.05); a *post-hoc* test showed mean bottom salinity varied significantly between zones 1 and 2 (−0.18), and 2 and 3 (0.15). The mean bottom salinity level increased from northern to middle sampling areas, and then decreased in the southern sampling areas. The mean bottom salinity varied significantly among eastern and western zones (*F* [2, 4829] = 373.5, *P* < 0.05); a *post-hoc* test showed mean bottom salinity varied significantly between zones 1 and 2 (−0.54), 1 and 3 (−1.07), and 2 and 3 (−0.52). Mean bottom salinity increased from eastern to western zones.

### Dissolved oxygen

The mean surface DO off the coast of New Jersey within 15 strata (areas: 12–26) during 1988 through 2015 ranged from 7.9 in 1995 to 8.98 in 1998 mg/L with a mean of 8.54 mg/L (±1.52 mg/L), and there was a weak positive association between the surface DO and time (Table 1). Overall, the mean DO increased about 0.009 mg/L per year or 0.09 mg/L per decade. Averaging over years, the mean monthly surface DO ranged from 7.17 in September to 10.27 mg/L in December. The surface DO varied significantly among months (*F* [11, 5080] = 608.75, *P* < 0.05).

The mean surface DO ranged from 8.20 in sampling area 24 to 9.06 mg/L in sampling area 14. The surface DO varied significantly among sampling areas (Table S2), and there was a strong negative association between the surface DO and the sampling area (Table S2). In general, the surface DO decreased from northern to southern sampling areas, but no pattern was evident for the sampling areas closest and furthest from shore (Table S1).

A paired *t*-test showed the annual bottom DO levels were significantly lower (*t* (5091) = 132.17; *P* <0.05) than the annual surface DO levels suggesting a strong water column stratification. The mean bottom DO off the coast of New Jersey within 15 strata (sampling areas: 12–26) during 1988 through 2015 ranged from 6.64 in 1988 to 8.35 mg/L in 1993 mg/L with a mean of 7.58 mg/L (±1.84 mg/L), and there was a weak positive association between the bottom DO and time (Table 1). The mean monthly bottom DO ranged from 5.55 in August to 9.87 mg/L in January. Averaging over years, the bottom DO varied significantly among months (*F* [11, 5081] = 1121.85, *P* < 0.05).

The mean bottom DO ranged from 7.09 in sampling area 16 to 8.13 mg/L in sampling area 24. The surface DO varied significantly among sampling areas (Table S2), and there was a strong positive association between the bottom DO and sampling area (Table S2). Segregating the sampling areas into zones (*See Methods*), the mean bottom DO varied significantly among northern and southern zones (*F* [2, 5090] = 54.78, *P* <0.05); a *post-hoc* test showed mean bottom DO varied significantly between zones 1 and 2 (−0.42), 1 and 3 (−0.69), and 2 and 3 (−0.27). The bottom DO generally increased from northern to southern sampling areas. The mean bottom DO also varied significantly among western and eastern zones (*F* [2, 4825] = 2.77, *P* < 0.05); a *post-hoc* test showed mean bottom DO varied significantly between zones 1 and 3 (0.15). The mean DO generally decreased from nearshore to offshore sampling areas.

### Marine fauna community

#### Annual and spatial dynamics

Over 20.4 million fish and invertebrates (1,338.3 mt) representing 214 (water temperature preference classified) species (*not including unidentified species*) were collected within 15 strata (areas: 12–26) off the coast of New Jersey from 1988 to 2015. Three marine fauna water temperature preference groups (coldwater-adapted, warmwater-adapted, and subtropic-adapted) were identified in the study area (Fig. S1). The total number of individuals collected ranged from 4.0 million (coldwater-adapted) to 6.8 million (subtropic-adapted). In each temperature preference category, three species represented the majority of the catch. The main coldwater-adapted species collected were longfin squid (*Loligo pealei*) (*n* = 2,225,975), Atlantic herring (*Clupea harengus*) (*n* = 544,032), and little skate (*Leucoraja erinacea*) (*n* = 316,356), while Atlantic butterfish (*Peprilus triacanthus*) (*n* = 2,873,138), scup (*Stenotomus chrysops*) (*n* = 1,318,569), and northern searobin (*Prionotus carolinus*) (*n* = 503,230) represented the warmwater-adapted group. Bay anchovy (*Anchoa mitchilli*) (*n* = 9,227,960), striped anchovy (*Anchoa hepsetus*) (*n* = 245,214), and Atlantic moonfish (*Vomer setapinnis*) (*n* = 38,691) denoted the subtropic-adapted group.

**Figure 2:**
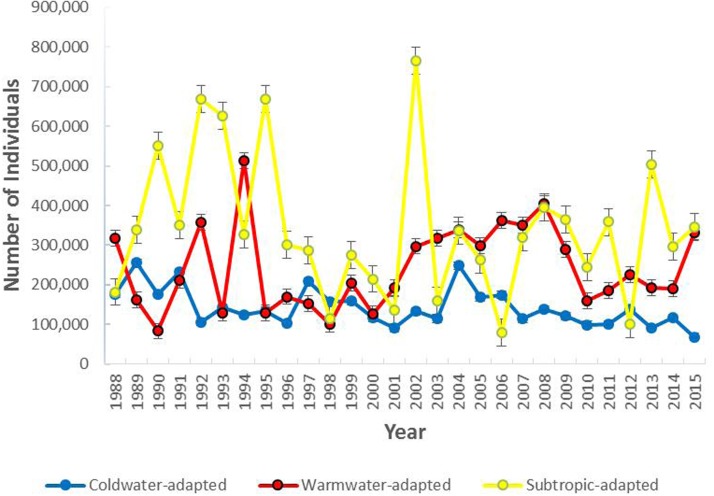
The total number of individuals collected per water temperature preference group within the study area (1988–2015). Error bars represent the standard error.

**Figure 3:**
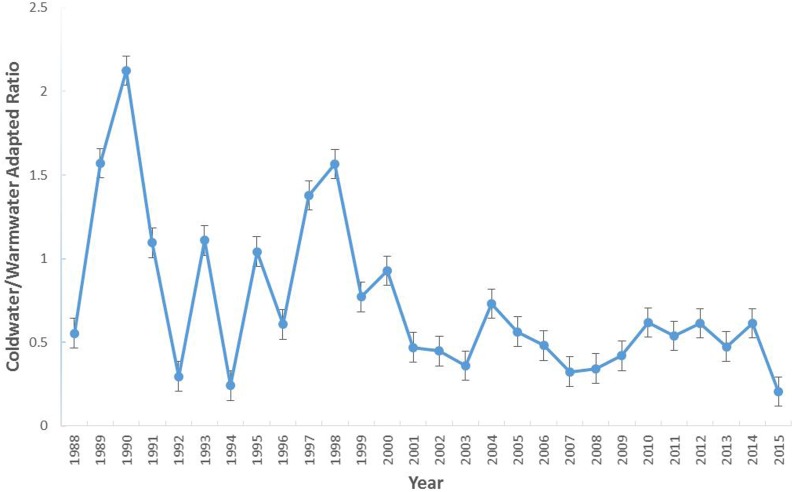
The coldwater to warmwater-adapted ratio of species collected within the study area (1988–2015). Error bars represent the standard error.

The most abundant water temperature preference group was the subtropic-adapted and the coldwater-adapted group was the least abundant group in the study area. The number of individuals collected per group varied over time with the coldwater-adapted group slightly decreasing since 2004 (Fig. 2). In terms of percent composition, coldwater-adapted group declined over time, but in some years (1989, 1990, 1991, 1993, 1995, 1997, 2006, and 2012) it was the second most dominant group (Fig. S2). In 1998, the coldwater-adapted group was the most abundant group. Overall, the coldwater to warmwater-adapted ratio declined over time, and there was a weak negative association between the coldwater: warmwater-adapted ratio and time (*F* [1, 26] = 10.71, *P* = 0.003; *r*^2^ = 29.2%) (Fig. 3)

Pooling all samples, the lowest (0.1784 individuals per m^2^) mean estimated abundance (# individuals/m^2^) was the warmwater-adapted group and highest (0.2340 individuals per m^2^) was the subtropic-adapted group (*F* [2, 100201] = 618.47, *P* < 0.05). The lowest mean estimated biomass (kg/m^2^) was the subtropic-adapted group and the highest was the coldwater-adapted group (*F* [2, 100201] = 687.35, *P* < 0.05).

**Figure 4:**
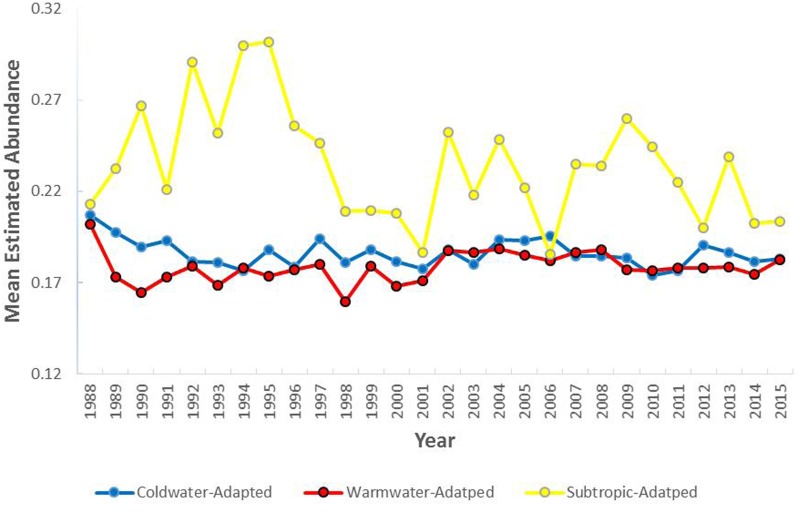
The mean annual estimated abundance (individuals/m^2^) by water temperature preference collected within the study area (1988–2015).

The mean estimated abundance of the coldwater-adapted group was consistently lower than the estimated abundance of the warmwater-adapted group over time (Fig. 4). The mean annual estimated abundance varied significantly by water temperature preference category (*F* [2, 100203] = 587.86, *P* < 0.05), time (*F* [27, 100203] = 11.93, *P* < 0.05), and the interaction between the water temperature group and time (*F* [54, 100203] = 9.33, *P* < 0.05). Overall, pooling the six 5-year time-series, the mean annual estimated abundance of the coldwater-adapted (*F* [5, 39039] = 8.82, *P* < 0.05) and subtropic-adapted (*F* [5, 7382] = 8.16, *P* < 0.05) groups decreased, and the warmwater-adapted group slightly increased (*F* [5, 39039] = 14.10, *P* < 0.05) over time.

The mean estimated abundance of the water temperature groups significantly varied by time (*F* [2, 100203] = 600.08, *P* < 0.05), space (*F* [14, 100203] = 3.93, *P* < 0.05), and the interaction between time and space (*F* [28, 100203] = 3.82, *P* < 0.05). The highest mean estimated abundance for the coldwater-adapted group was in the northern sampling areas (sampling areas 13, 14, and 12), while the highest mean estimated abundance for the warmwater-adapted (sampling areas 20, 18, and 19) and subtropic-adapted (sampling areas 22, 20, and 19) groups was in mid and southern sampling areas.

Similarly, the mean annual estimated biomass varied significantly by water temperature preference category (*F* [2, 100203] = 705.8, *P* < 0.05), time (*F* [27, 100203] = 6.49, *P* < 0.05), space (*F* [14, 100203] = 2.92, *P* < 0.05), and the interaction between time and space (*F* [54, 100203] = 3.79, *P* < 0.05). The mean annual estimated biomass for all three water temperature preference groups (coldwater-adapted [*F* [5, 39039] = 38.84, *P* < 0.05]; warmwater-adapted [*F* [5, 39039] = 44.03, *P* <0.05]; and subtropic-adapted [*F* [5, 7382] = 10.35, *P* < 0.05] increased with time, but the mean annual estimated biomass of the subtropic-adapted group increased the most and coldwater-adapted group increased the least. The interaction pattern between the mean annual estimated biomass and space was less clear than the mean annual estimated abundance and time; there was no pattern in the mean annual estimated biomass of the coldwater-adapted [sampling areas 18, 13, and 23], warmwater-adapted [sampling areas 20, 12, and 19], and subtropic-adapted [sampling areas 18, 12, and 22] groups.

Pooling the data (1988–2015), GLMs showed that year and sampling area were significant predictors of the total number and estimated abundance of the coldwater and warmwater-adapted groups. The total number and estimated abundance of the subtropic-adapted group were significantly predicted by the year, month, and sampling area, whereas the estimated abundance was only significantly predicted by the year and sampling area (Tables 2–4).

**Table 2:** General Linear Model and associated ANOVA Type III Sums of Squares. Coldwater-adapted species Temperature Preference. Five-yr Time-series (1988–2015). CW, Coldwater-adapted species.

**Dependent variable**	**Source**	**Sum of squares**	*Df*	**Mean square**	*F*-ratio	*P*-value	**Fitted model**	*R*^2^
Total number (Coldwater-adapted)	Time Series (CW)	11.3453	1	11.3453	7.99	0.0047	√√ Total Number (CW) = 6.3662–2.13748E–7*Time Series (CW) + 0.0192003*Month (CW) - 0.0104509*Area (CW)	0.41%
	Month (CW)	136.547	1	136.547	96.15	0.0000		
	Area (CW)	74.3637	1	74.3637	52.36	0.0000		
	Residual	55435.6	39036	1.42011				
	Total (corrected)	55663.8	39039					
Estimated abundance (Coldwater-adapted)	Time series (CW)	0.0292921	1	0.0292921	2.38	0.1229	√√ Estimated Abundance (CW) = 0.413077–1.0861E–8*Time Series (CW) + 0.00168064*Month (CW)–0.000971917*Area (CW)	0.36%
	Month (CW)	1.0462	1	1.0462	85.01	0.0000		
	Area (CW)	0.643144	1	0.643144	52.26	0.0000		
	Residual	480.408	39036	0.0123068				
	Total (corrected)	482.161	39039					
Estimated biomass (coldwater-adapted)	Time Series (CW)	0.0336629	1	0.0336629	6.63	0.0100	√√ Estimated Biomass (CW) = −0.0790099 + 1.16431E–8*Time series (CW) - 0.00145997*Month (CW) − 0.00139156*Area (CW)	1.06%
	Month (CW)	0.789501	1	0.789501	155.47	0.0000		
	Area (CW)	1.31841	1	1.31841	259.62	0.0000		
	Residual	198.236	39,036	0.00507829				
	Total (corrected)	200.357	39,039					

**Table 3:** General Linear Model and associated ANOVA Type III Sums of Squares. Warmwater-adapted species Temperature Preference. Five-yr Time-series (1988–2015). WW, Warmwater-adapted species.

**Dependent variable**	**Source**	**Sum of squares**	*Df*	**Mean square**	*F*-ratio	*P*-value	**Fitted model**	*R*^**2**^
Total number (Warmwater-adapted)	Time Series (WW)	60.3819	1	60.3819	40.24	0.0000	√√ Total Number (WW) = −8.22361 + 4.92926E-7*Time Series (WW) + 0.0550105*Month (WW) −0.00515665*Area (WW)	1.66%
	Month (WW)	922.387	1	922.387	614.75	0.0000		
	Area (WW)	18.0308	1	18.0308	12.02	0.0005		
	Residual	58,570.3	39,036	1.50042				
	Total (corrected)	59,559.4	39,039					
Estimated abundance (warmwater-adapted)	Time Series (WW)	0.780301	1	0.780301	60.04	0.0000	√√ Estimated Abundance (WW) = −0.967161 + 5.6035E-8*Time Series (WW) + 0.00501172*Month (WW)–0.000490182*Area (WW)	1.64%
	Month (WW)	7.65589	1	7.65589	589.07	0.0000		
	Area (WW)	0.162928	1	0.162928	12.54	0.0004		
	Residual	507.331	39036	0.0129965				
	Total (corrected)	515.812	39,039					
Estimated biomass (warmwater-adapted)	Time Series (WW)	0.800626	1	0.800626	164.43	0.0000	√√ Estimated Biomass (WW) = −1.0566 + 5.67601E–8*Time Series (WW) + 0.00400921*Month (WW) + 0.000216194*Area (WW)	2.91%
	Month (WW)	4.89938	1	4.89938	1006.22	0.0000		
	Area (WW)	0.0316933	1	0.0316933	6.51	0.0107		
	Residual	190.069	39,036	0.00486908				
	Total (corrected)	195.747	39,039					

### Segregated spatial dynamics

#### North/south spatial dynamics

Subtropic-adapted species were the most abundant category and coldwater-species were the least abundant category in every north/south zones; north/south zones were designated as the following: north (sampling areas 12–17); mid (sampling areas 18–23), and south (sampling areas 24–26). The total number of coldwater and warmwater-adapted species decreased from north to south, but the mean annual estimated abundance of coldwater (*F* [2, 40368] = 0.21, *P* = 0.81) and warmwater-adapted groups (*F* [2, 40368] = 2.35, *P* = 0.0957) did not vary significantly among north and south zones.

**Table 4:** General linear model and associated ANOVA Type III sums of squares. Subtropical-adapted species temperature preference. Pooled 5-yr time-series (1988–2015). ST, Subtropical-adapted species.

**Dependent variable**	**Source**	**Sum of squares**	*Df*	**Mean square**	*F*-ratio	*P*-value	**Fitted model**	*R*^**2**^
Total number (subtropic-adapted)	Time series (ST)	135.366	1	135.366	19.51	0.0000	√√ Total Number (ST) = 34.9198–0.00000161118*Time Series (ST) + 0.0682713*Month (ST) - 0.0374255*Area (ST)	0.88%
	Month (ST)	139.303	1	139.303	20.08	0.0000		
	Area (ST)	168.857	1	168.857	24.34	0.0000		
	Residual	5,1187.0	7,379	6.93685				
	Total (corrected)	5,1642.8	7,382					
Estimated abundance (subtropic-adapted)	Time series (ST)	0.992579	1	0.992579	16.37	0.0001	√√ Estimated Abundance (ST) = 3.01224–1.37966E–7*Time Series (ST) + 0.00626256*Month (ST)–0.00351368*Area (ST)	0.83%
	Month (ST)	1.17216	1	1.17216	19.33	0.0000		
	Area (ST)	1.48837	1	1.48837	24.54	0.0000		
	Residual	447.466	7,379	0.0606405				
	Total (corrected)	451.214	7,382					
Estimated biomass (subtropic-adapted)	Time series (ST)	0.203569	1	0.203569	42.11	0.0000	√√ Estimated Biomass (ST) = -1.19149 + 6.24808E–8*Time Series (ST)–0.0000933727*Month (ST) + 0.00143838*Area (ST)	1.23%
	Month (ST)	0.00026057	1	0.00026057	0.05	0.8164		
	Area (ST)	0.249422	1	0.249422	51.59	0.0000		
	Residual	35.6733	7,379	0.00483444				
	Total (corrected)	36.1178	7,382					

A two-way ANOVA showed the mean annual estimated abundance of coldwater-adapted species varied significantly by time and space (*F* [54, 40368] = 1.87, *P* = 0.0001). The mean annual estimated abundance of coldwater species was highest in zone 1 during a few years (1994, 1997, 2004–2006, 2008, 2011, and 2012). In contrast, the mean annual estimated abundance of coldwater species was highest in zone 3 during 1988-1990, 1992, and 2003.

The mean annual estimated abundance of warmwater species also varied significantly by time and space, including the interaction between time and space (*F* [42, 40368] = 2.71, *P* < 0.05). The highest mean annual estimated abundance in Zone 1 occurred in 1994, 2003, and 2004. In Zone 3, the highest mean annual estimated abundance occurred in 1988, 1992 and 1999.

The mean annual estimated abundance of subtropic-adapted species varied significantly by time (*F* [27, 7474] = 1.74, *P* = 0.01) and space (*F* [2, 7471] = 4.54, *P* = 0.01). The highest mean annual estimated abundance of subtropic-adapted species was in Zone 2 (*F* [2, 7471] = 4.15, *P* = 0.0158) followed by Zone 1 and Zone 3. The highest mean annual estimated abundance in Zone 1 occurred in 1997, 2001, 2003, 2004, 2007, 2008, 2011, and 2015. In Zone 3, the highest mean annual estimated abundance occurred in 1990 and 2005.

The estimated biomass of coldwater-adapted species peaked in Zone 1 during 2000 and 2007, but it did not vary significantly by time (*F* [27, 40368] = 0.58, *P* = 0.95) or space (*F* [2, 40368] = 2.21, *P* = 0.11). Similarly, the estimated biomass of warmwater-adapted species peaked in Zone 2 during 2005, but it did not vary significantly by time *F* [27, 40368] = 0.85, *P* = 0.65) or space (*F* [2, 40368] = 0.61, *P* = 0.82). The estimated abundance of subtropic-adapted species peaked in Zone 3 during 2012, but it did not vary significantly by time *F* [27, 7471] = 0.89, *P* = 0.62) or space (*F* [2, 7471] = 0.86, *P* = 0.42). The estimated biomass of subtropic-adapted species increased in Zone 2 during 2012 through 2015.

### West/east spatial dynamics

The mean annual estimated abundance of subtropic-adapted species was highest and the coldwater-adapted species were lowest in every west/east zone; west/east zones were designated as the following: west (sampling areas 12, 15, 18, 21, and 24); mid (sampling areas 13, 16, 19, 22, and 25), and east (sampling areas 14, 17, 20, 23, and 26). The mean annual estimated abundance of subtropic-adapted species decreased from west (Zone 1) to east (Zone 2) Zones, and coldwater and warmwater-adapted species increased from west to east zones.

The mean annual estimated abundance of coldwater-adapted species varied significantly by time (*F* [27, 40368] = 2.07, *P* = 0.0009) and space (*F* [2, 40368] = 4.81, *P* = 0.008). The mean annual estimated abundance in Zone 1 was the highest in 1993 and 2004. In Zone 3, the mean annual estimated abundance peaked in 1990 and 2011.

The mean annual estimated abundance of warmwater-adapted species varied significantly by time (*F* [27, 40368] = 2.09, *P* = 0.002) and interaction between time and space (*F* [42, 40368] = 1.64, *P* = 0.005). The mean annual estimated abundance peaked in Zone 3 during 1992. In Zone 2, the mean annual estimated abundance peaked in 1994, 1996, 1999, 2002, 2004, and 2006.

The mean annual estimated abundance of subtropic-adapted species varied significantly over time (*F* [27, 7471] = 1.90, *P* = 0.003). The mean annual estimated abundance in Zone 1 peaked in 1990, 2000, 2002, and 2014. The mean annual estimated abundance in Zone 2 peaked 1992, 1995, 2008, and 2013. In Zone 3, the mean annual estimated abundance peaked in 1993, 2009, and 2011.

Overall, the highest mean annual estimated biomass was warmwater-adapted species, but there was no evidence to suggest the mean annual estimated biomass among temperature preference categories significantly changed from western to eastern areas.

The mean annual estimated biomass of coldwater-adapted species did not vary significantly by time (*F* [27, 40368] = 1.21, *P* = 0.21) or space (*F* [2, 40368] = 2.90, *P* = 0.0552). The mean annual estimated biomass in Zone 3 peaked in 2000, and in Zone 2 it peaked during 2007; the mean annual estimated biomass in Zone 1 was relatively low throughout the time period.

Similarly, the mean annual estimated biomass of warmwater-adapted species did not vary significantly by time (*F* [27, 40368] = 1.09, *P* = 0.35) or space (*F* [2, 40368] = 0.35, *P* = 0.89). In Zone 1, the mean annual estimated biomass peaked in 2012, and peaked in Zone 2 in 2005. In Zone 3, the mean annual estimated biomass peaked in 1992, 2001, and 2007.

The mean annual estimated biomass of subtropic-adapted species did not vary significantly by time (*F* [27, 7471] = 0.31, *P* = 0.99) or space (*F* [2, 7471] = 0.03, *P* = 0.97). In Zone 1, the estimated biomass peaked in 1991, and increased from 2012 to 2015. In Zone 2, the mean annual estimated biomass peaked in 2004 and 2012. The mean annual estimated biomass in Zone 3 was relatively low during most of the years, but it peaked in 1997 and 2014.

## Discussion

### Physicochemical conditions

Identifying the annual and seasonal variability in the oceanic conditions and the response of community and component populations within the ecosystem is critical for predicting long-term community dynamics, trends, and evaluating disturbance. Given the broad oceanographic hydrodynamics off New Jersey (Kohut, Glenn & Chant, 2004), the water temperature, salinity, and DO levels significantly varied over time and space in the study area. The mean oceanic conditions were highly variable over the 28-year period, but various alternating or cyclic patterns were evident, along with increasing or decreasing trends. In general, water temperature increased and the salinity decreased over time. The rising water temperature and falling salinity trends echoed previous studies for the region (Howell & Auster, 2012; Geiger et al., 2013; Thomas et al., 2017). The trend rates for mean (0.06 °C per year) and maximum (0.02 °C per year) surface water temperature were similar to published rates (Thomas et al., 2017).

The nearshore waters off New Jersey are a dynamic hydrological system influenced by summer stratification and winter mixing, which are associated with the prevailing wind and buoyancy factors (Glenn et al., 2004; Kohut, Glenn & Chant, 2004). Northeast wind often causes downwelling, while southwest wind causes upwelling (Kohut, Glenn & Chant, 2004). The physicochemical conditions varied not only by year and season, but among specific sampling areas within the study area. Besides the southeast corner of the study area (sampling areas 21 and 24), water temperature (surface and bottom), salinity (surface), and DO increased from northern to southern sampling areas, and salinity increased from western to eastern sampling areas. Bottom salinity decreased from middle to southern areas, and the surface DO decreased from northern to southern areas. Bottom DO increased from northern to southern sampling areas, and decreased from eastern to western sampling areas.

Upwelling and downwelling off New Jersey are frequent events given the wide continental shelf and gently sloping topography; the continental shelf extends about 200 km off the coast of New Jersey (Song, Haidvogel & Glenn, 2001; Kohut, Glenn & Chant, 2004). It is possible the relatively warmer water temperature and higher salinity in sampling areas 21 and 24 was influenced by the nearby underlying topography (i.e., topographic bump and oblique sand ridges) or seasonal upwelling events. The nearshore waters (∼40 m) are more stratified than the offshore waters in June because of the influence of lower salinity water from the Hudson River (Schofield et al., 2008), which could be causing the decreasing salinity trend off the New Jersey coast, noted here. The warmwater period in the MAB is longer now than in the past and this environmental change has influenced the stratified period (June–September) thereby altering the timing of spring and fall phytoplankton blooms. Over the past decade, the warming period is beginning earlier each year (Thomas et al., 2017).

The surface and bottom DO varied significantly over time and the positive association between DO and time was explained adequately by regression, but a low correlation coefficient value (0.08) indicated a weak association. It is difficult to explain why the DO slightly increased (0.009 mg/L per year) with time given research has shown several recurrent hypoxia events (1994, 1996, and 2001) occurring along the southern New Jersey coast (Glenn et al., 2004). Hypoxia is relatively common along the New Jersey coast, but these events are somewhat short-term and related to coastal upwelling, which sometimes occurs in summer when the wind is from the southwest (Glenn et al., 2004). The hypoxia centers (∼150 km^2^) are spatially isolated in duration (∼1 week), frequency (∼5 times in 9 years), and space (Barnegat Inlet, Mullica River Estuary, and Townsend/Hereford Inlets). The hypoxia locations are downstream of a series of topographic highs associated with ancient river deltas in the southern waters off New Jersey. The most significant upwelling events occurred after the most severe cooling seasons in 1994, 1996, and 2001 (Glenn et al., 2004). Researchers hypothesized that severe cooling seasons often causes colder and larger Cold Pools, which produce more significant summer upwelling events by summertime wind-driven forces. Upwelling also depends upon wind, precipitation, and storm frequency. Given that water temperature is increasing in the study area, it is possible that summer upwelling events could be less severe than in the past, which is reducing the magnitude of hypoxia events in specific areas within the study area. This hypothesis might explain the slight increase in DO over time. Then again, it might be related to the ongoing water quality improvements (less pollution) in nearby New York and New Jersey waterbodies (HydroQual, 2010).

### Marine fauna community

The nearshore coastal waters off New Jersey provide year-round and seasonal habitat for three temperature preference groups (subtropic-adapted, warmwater-adapted, and coldwater-adapted), which complemented previous findings in the MAB (Wood, Collie & Hare, 2009; Howell & Auster, 2012). However, different than Howell & Auster (2012), subtropic-adapted species were the most abundant and coldwater-adapted species were the least abundant in the study area. Bay anchovy was the most abundant subtropic-adapted species and butterfish was the most abundant warmwater-adapted species. These findings did not agree with Howell & Auster (2012) who reported more coldwater-adapted species than warmwater-adapted and subtropic-adapted species in Long Island Sound. New Jersey waters are not only located south of Long Island Sound, but anecdotal information from local fishermen suggest warmwater eddies and whorls spinning off the Gulf Stream Current can provide habitat to subtropic-adapted marine fauna, which could explain why there were more subtropic-adapted species in New Jersey than New York.

The estimated abundance of warmwater-adapted species is increasing, coldwater and subtropic-adapted species is decreasing, and the coldwater to warmwater-adapted ratio is decreasing over time. This ongoing pattern for marine fauna seems to be becoming more common in the MAB (Nye et al., 2009; Wood, Collie & Hare, 2009; Howell & Auster, 2012), and throughout the world (e.g.,  Polovina et al., 2011). Most researchers attribute this shift in species distribution and composition to climate variability (Cheung et al., 2011; Jang et al., 2011; Polovina et al., 2011; Brander, 2013). The abundance of warmwater-adapted species is also increasing with time in Narragansett Bay (Wood, Collie & Hare, 2009). In nearby Long Island Sound, Howell & Auster (2012) reported a shift from a coldwater to warmwater-adapted dominated species community, and an increase in subtropic-adapted species over a 25-year duration (1984–2008). It is possible the present study did not show this same trend for subtropic-adapted species because the environmental conditions off New Jersey are more variable than in the Long Island Sound (a semi-enclosed estuary) given the frequency of upwelling and downwelling events (Glenn et al., 2004; Kohut, Glenn & Chant, 2004). Though the mean water temperature is rising, the frequency of upwelling/downwelling is also increasing in the MAB (Kohut, Glenn & Chant, 2004), which could explain the decline in estimated abundance of subtropic-adapted species over time. It is also possible subtropic-adapted species cannot tolerate abrupt changes in water temperatures caused by these events. Thus, these oceanographic events could be negatively impacting some subtropic-adapted species since the influx of cold water/warm water (±1 −4 °C) can often occur in a short (∼3 or 4 weeks) period (Kohut, Glenn & Chant, 2004).

The highest estimated abundance of coldwater-adapted species was in northern sampling areas (12–17), and the highest estimated abundance for warmwater and subtropic-adapted species was in mid and southern sampling areas (18–26). The total number of coldwater and warmwater-adapted species decreased from north to south, but the estimated abundance did not vary significantly among the study area. Explaining the distribution of species is not straightforward in terms of water temperature (Wood, Collie & Hare, 2009), as it appears to vary by time, space and season given the oceanographic dynamics in the study area. Despite this variable pattern, the estimated abundance of coldwater-adapted species was generally higher in the southern sampling areas (24–26) during earlier years (1990s) and higher in the northern sampling areas (12–14) during later years (2000s). The estimated abundance of warmwater-adapted species was more variable, but it was higher in the southern sampling areas during a few earlier years (1990s) and higher in the northern sampling areas during a few later years (2000s). The estimated abundance of subtropic-adapted species was highest in the middle sampling areas (18–23), followed by the northern (12–17) and southern sampling (24–26) areas. In general, the highest estimated abundance was higher in the southern sampling areas (24–26) during a few earlier years, and higher in the northern sampling areas (12–17) during later years. In some ways, the findings suggest the distribution of species, based on their temperature preference, is currently in a transitional phase; the period when some individuals can still tolerate the mean water temperature. For instance, the water temperature preference classification for this study was based on the mean preferred water temperature, which means 50 percent of the individuals can tolerate either a lower or higher water temperature. Assuming the water temperature continues to rise with time, then a full transition from a coldwater dominated community to a warmwater community will occur over time.

The estimated abundance of coldwater and warmwater-adapted species increased from nearshore to offshore and subtropic-adapted species decreased from nearshore (12, 15, 18, 21, 24) to offshore (14, 17, 20, 23, 26) sampling areas. The increase in the estimated abundance of warmwater-adapted species from nearshore to offshore sampling areas suggests the environmental conditions are ideal for a geographical range expansion within the study area given the rising water temperature. In general, the estimated abundance of coldwater-adapted species was higher in the nearshore sampling areas in a few earlier years (1990s) and higher in the offshore sampling areas in a few later years (2000s). These findings suggest the distribution of coldwater and warmwater-adapted species is shifting north, which agrees with other findings in the MAB (Nye et al., 2009; Howell & Auster, 2012).

The estimated abundance for all three temperature preference categories has changed over time. The estimated abundance of subtropic-adapted species decreased from nearshore to offshore sampling areas, and coldwater and warmwater-adapted species increased from nearshore to offshore sampling areas. Despite these general patterns, inter-annual patterns were challenging to decipher given their high annual population and habitat selection variability. For instance, coldwater-adapted species had high abundance in the nearshore sampling areas during 1993 and 2004, and high abundance in the offshore zone during 1990 and 2011. The findings partially support the hypotheses that the estimated abundance and biomass of warmwater and subtropic-adapted assemblages increased from offshore to nearshore sampling areas, and the estimated abundance and biomass of coldwater-adapted assemblage increased from nearshore to offshore sampling areas with time. Overall, it appears that biomass trends are idiosyncratic to specific areas within the overall study area, but these findings could be more related to how the sampling was spatially segregated rather than to biological reasons.

The estimated biomass for all three water temperature preference categories is increasing with time in the study area; however, the estimated biomass of coldwater-adapted species is increasing the most even though they are decreasing in abundance throughout the study area. Larger individuals seem to be replacing smaller individuals, or less abundant larger individuals are more common in the study area, such as bullnose sting ray (*Dasyatis sayi*). It is difficult to explain why the estimated biomass of subtropic-adapted species is increasing the most since their estimated abundance is decreasing with time. Smaller or younger (juvenile life-stage) coldwater-adapted individuals could be declining, moving away, or maybe the larger less abundant subtropic-adapted species are moving into the study area to feed. Actually, it is possible that juvenile/sub-adults are moving offshore to deeper colder waters, while adults are moving nearshore in pursue of prey. Another potential explanation is that rising water temperature is causing coldwater-adapted species to grow faster (Duffy et al., 2016). It is often difficult to explain or generalize the response of fish populations to climate change given the number of influential factors and individual species broad responses (Rijnsdorp et al., 2009).

The estimated biomass of coldwater and warmwater-adapted species was influenced by month and sampling area, whereas subtropic-adapted species were generally more influenced by the sampling area. This observation seems reasonable since the estimated abundance of most species in the coldwater and warmwater-adapted groups is correlated with season (i.e., water temperature). However, it was somewhat surprising that the subtropic-adapted group was not influenced by season, but it is possible that certain sampling areas have ideal and less fluctuating water temperatures given the oceanic dynamics (upwelling/downwelling and eddies) in the study area (Kohut, Glenn & Chant, 2004). The total number and estimated abundance of coldwater-adapted and warmwater-adapted species in recent years (2013–2015) were influenced more by month and sampling area than the previous years (2008–2012) suggesting the seasonal water temperature could be rising and falling faster depending on the sampling area. Actually, the warming period is occurring earlier and lasting longer (Thomas et al., 2017), which could explain the decreasing and increasing abundance in coldwater-adapted and warmwater-adapted groups, respectively.

## Conclusion

The nearshore waters off New Jersey provide habitat for a variety of marine fauna, including various warmwater and subtropic-adapted species. Overall, the findings indicate the abundance and distribution of coldwater, warmwater, and subtropic-adapted species is changing with time. In particular, the coldwater-warmwater-adapted ratio is declining with time, which appears to be linked to the rising water temperature. Despite these findings, it should be acknowledged this study was limited in scope in terms of examining wide-ranging potential disturbances or stressors (i.e., fishing impacts). Thus, future researchers should consider evaluating other known stressors besides the environmental conditions, such as fisheries (commercial and recreational), habitat loss, and poor water quality. Although researchers have investigated some of these stressors individually, and the impacts of individual stress on specific species, it is recommended future research examine various stressors in synchronicity since they are not independent of each other; cumulative effects are known to impact natural resources.

##  Supplemental Information

10.7717/peerj.7927/supp-1 Table S1 The mean annual physicochemical conditions within specific sampling area (1988–2015)

10.7717/peerj.7927/supp-2 Table S2 The annual spatial physicochemical conditions in the study area (1988–2015)

10.7717/peerj.7927/supp-3 Figure S1 The total number of individuals collected per water temperature preference category in the study area (1988–2015)

10.7717/peerj.7927/supp-4 Figure S2 Percent composition of individuals collected by water temperature preference category within the study area (1988–2015). C, coldwater-adapted, W, warmwater-adapted, and S, subtropic-adapted

## Additional Information and Declarations

### Data Availability

The following information was supplied regarding data availability: The raw data is available through the New Jersey Department of Environmental Protection (NJDEP), Division of Fish and Wildlife by contacting Linda M. Barry, Assistant Biologist at the Marine Fisheries Administration Nacote Creek Research Station (telephone: 1-609-748-2020; email: Linda.Barry@dep.state.nj.us). The NJDEP, like most state and federal agencies, requires researchers to formally request and sign a Memorandum of Understanding (MOU) before they can receive any data collected by the state. The MOU establishes a framework of how the data will be analyzed and used given all the particulars of fisheries independent monitoring programs.
