# Supplementary material for: Spatio-temporal patterns of the oceanic conditions and nearshore marine community in the Mid-Atlantic Bight (New Jersey, USA)
Source: PeerJ. 2019 Oct 21;7:e7927. doi: 10.7717/peerj.7927 (PMC6812665; doi:10.7717/peerj.7927)
Supplement: Table S1 [file peerj-07-7927-s001.docx]

| **Sampling Area** | **Surface Water Temperature** | **Bottom Water Temperature** | **Surface Salinity** | **Bottom Salinity** | **Surface DO** | **Bottom DO** |
| --- | --- | --- | --- | --- | --- | --- |
| 12 | 13.8067 | 12.6383 | 28.1299 | 30.3325 | 8.97390 | 7.46609 |
| 13 | 13.9227 | 11.3925 | 28.5114 | 31.6522 | 8.95303 | 7.09992 |
| 14 | 14.2203 | 10.6697 | 29.8132 | 32.0772 | 9.06167 | 7.20421 |
| 15 | 14.6226 | 13.2393 | 30.3907 | 31.1852 | 8.67297 | 7.4245 |
| 16 | 14.808 | 12.1511 | 30.5195 | 31.6326 | 8.80331 | 7.08529 |
| 17 | 15.0519 | 10.8999 | 31.0661 | 32.1597 | 8.64415 | 7.17957 |
| 18 | 14.8423 | 13.8549 | 30.9141 | 31.2409 | 8.27636 | 7.76678 |
| 19 | 15.0082 | 12.72 | 31.0381 | 31.7288 | 8.46681 | 7.51636 |
| 20 | 15.2472 | 11.5188 | 31.5304 | 32.2017 | 8.38812 | 7.55482 |
| 21 | 14.3163 | 13.5188 | 31.2977 | 31.4676 | 8.40565 | 7.97236 |
| 22 | 15.2959 | 13.4806 | 31.4918 | 31.7311 | 8.32289 | 7.82684 |
| 23 | 15.5203 | 11.9391 | 31.7639 | 32.3242 | 8.31835 | 7.69609 |
| 24 | 14.3215 | 14.2524 | 30.5373 | 30.7914 | 8.19962 | 8.12590 |
| 25 | 15.2639 | 13.5825 | 31.3253 | 31.7725 | 8.28575 | 7.97881 |
| 26 | 15.007 | 12.0479 | 31.4876 | 32.2082 | 8.40753 | 7.92440 |

**Table S1**. The mean annual physicochemical conditions within specific sampling area (1988−2015).
