# Supplementary material for: Spatio-temporal patterns of the oceanic conditions and nearshore marine community in the Mid-Atlantic Bight (New Jersey, USA)
Source: PeerJ. 2019 Oct 21;7:e7927. doi: 10.7717/peerj.7927 (PMC6812665; doi:10.7717/peerj.7927)
Supplement: Table S2 [file peerj-07-7927-s002.docx]

**Table S2**. The annual spatial physicochemical conditions in the study area (1988−2015).

| **Environmental Parameter** | **Hypothesis Test for Interannual Variation** | **Test Results** | **Regression Model for Spatial Trend** | **Regression Equation** | **F-test** | **r^2^** |
| --- | --- | --- | --- | --- | --- | --- |
| Surface Water Temperature | ANOVA | F [14, 5081] = 1.98,  *P* = 0.016 | Linear | Surface Water Temperature = 13.3292 + 0.0747982*Area | *F* [1, 13] = 13.95, *P =* 0.0025 | 51.8% |
| Bottom Water Temperature | ANOVA | F [14, 5081] = 11.48,  *P* < 0.05 | Linear | Bottom Water Temperature = 10.5857 + 0.102179*Area | *F* [1, 13] = 2.65, *P* = 0.1276 | 16.9% |
| Surface Salinity | ANOVA | F [14, 5081] = 94.31,  *P* < 0.05 | Linear | Surface Salinity = 27.0243 + 0.191061*Area | *F* [1, 13] = 47.99, *P <* 0.05 | 78.7% |
| Bottom Salinity | ANOVA | F [14, 5082] = 72.5,  *P* < 0.05 | Linear | Bottom Salinity = 30.8296 + 0.0423243*Area | *F* [1, 13] = 2.47, *P =* 0.14 | 15.9% |
| Surface Dissolved Oxygen | ANOVA | F [14, 5077] = 10.49,  *P* < 0.05 | Linear | Surface DO = 9.59736 - 0.0553696*Area | *F* [1, 13] = 61.62, *P <* 0.05 | 82.6% |
| Bottom Dissolved Oxygen | ANOVA | F [14, 5078] = 9.87,  *P* < 0.05 | Linear | Bottom DO = 6.382 + 0.0634807*Area | *F* [1, 13] = 29.38, *P* = 0.0001 | 69.3% |
