# Supplementary figures and images for: Spatio-temporal patterns of the oceanic conditions and nearshore marine community in the Mid-Atlantic Bight (New Jersey, USA)
Source: PeerJ. 2019 Oct 21;7:e7927. doi: 10.7717/peerj.7927 (PMC6812665; doi:10.7717/peerj.7927)

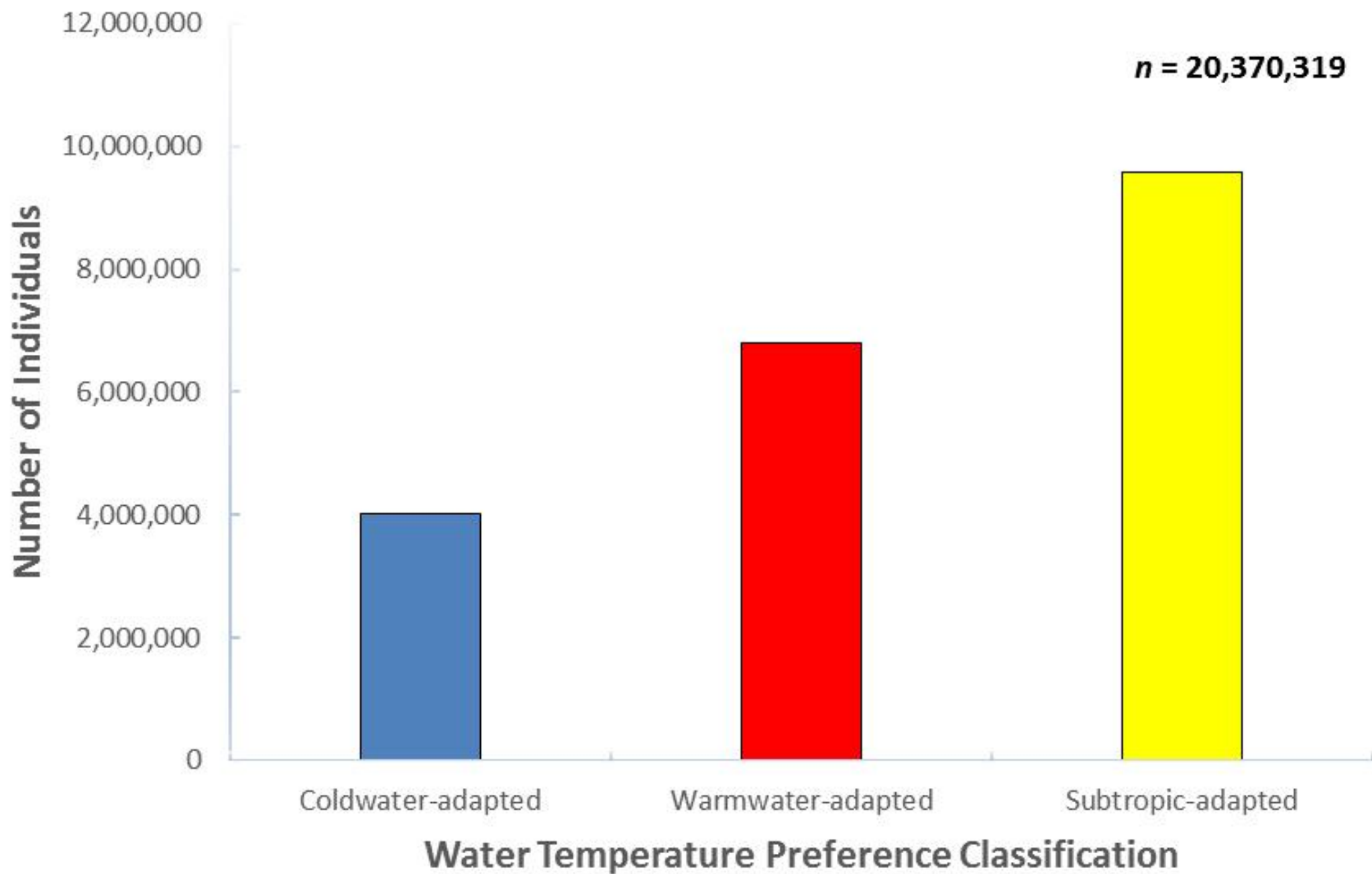

Supplement: Figure S1 [file peerj-07-7927-s003.pdf]

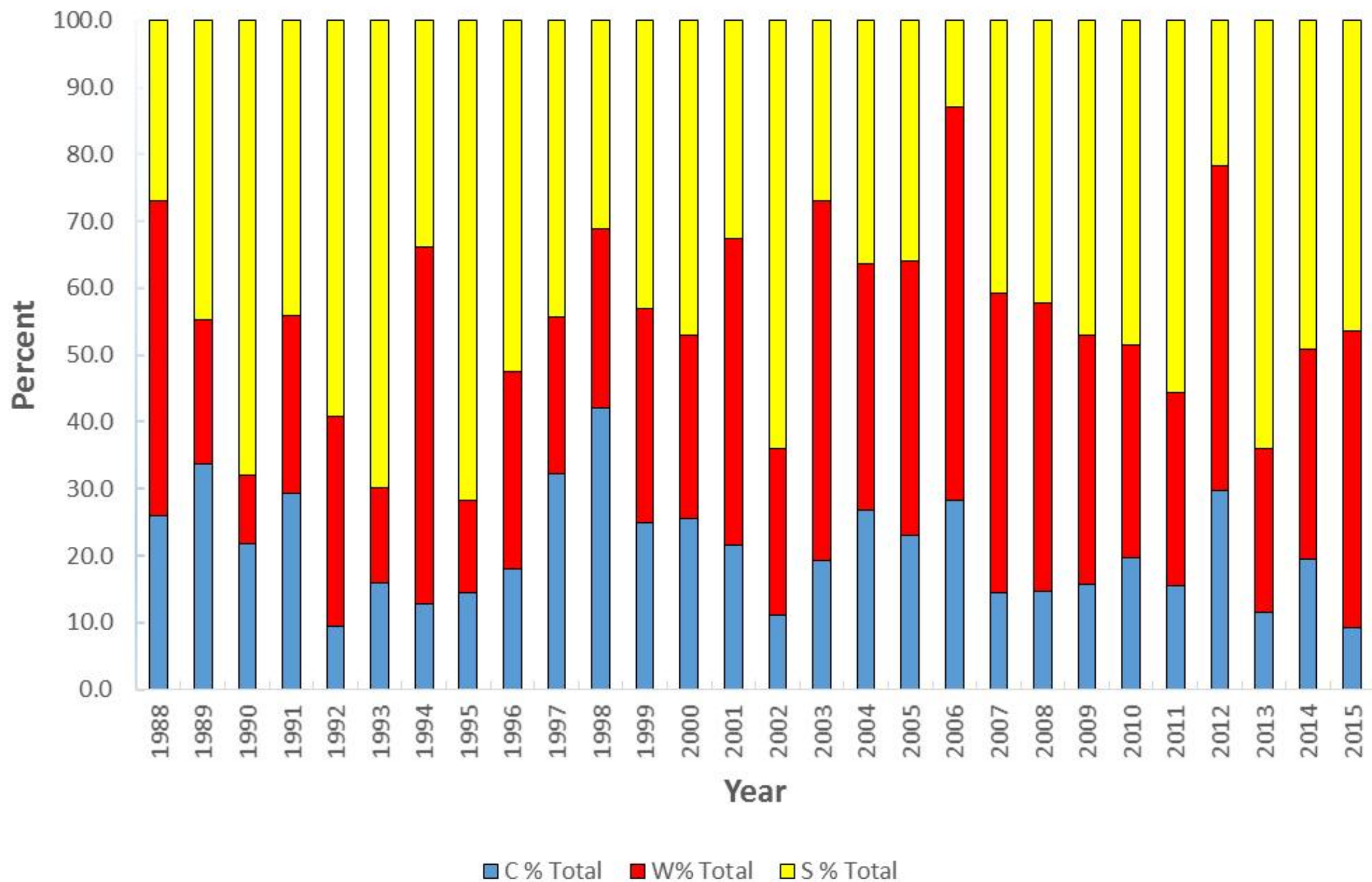

Supplement: Figure S2 [file peerj-07-7927-s004.pdf]
